# Applications of Graphene-Based Materials in Sensors

**DOI:** 10.3390/s20113196

**Published:** 2020-06-04

**Authors:** Miguel Hernaez

**Affiliations:** School of Engineering, Faculty of Science, University of East Anglia, Norwich Research Park, Norwich NR4 7TJ, UK; m.hernaez@uea.ac.uk

**Keywords:** graphene, graphene oxide, reduced graphene oxide, sensors, physical sensors, chemical sensors, gas sensors, biosensors

## Abstract

This Special Issue compiles a set of innovative developments on the use of graphene-based materials in the fabrication of sensors. In particular, these contributions report original studies on a wide variety of sensors, such as gas sensors for NO_2_ or NH_3_ detection, antibody biosensors or mass sensors. All these devices have one point in common: they have been built using graphene-based materials like graphene, graphene oxide, reduced graphene oxide, inkject printing graphene, graphene-based composite sponges, graphene screen-printed electrodes or graphene quantum dots.

## 1. Introduction

Graphene has become the most explored material since Novoselov and Geim (Nobel Prize winners for Physics in 2010) achieved its isolation in 2004. The exceptional properties of graphene have attracted the attention of the scientific community from different research fields. Graphene is a monolayer of hexagonally arrayed sp^2^-bonded carbon atoms, extremely sensitive to the external environment. Therefore, sensing is one of the many fields that can benefit from the use of this exciting material.

However, the development of a method for the production of high-quality graphene in large quantities at a competitive cost is essential to further exploit its full potential. For that reason, the use of graphene-based materials as graphene oxide (GO) and reduced graphene oxide (rGO), among others, has gained widespread consideration, as a compromise between the interesting properties of graphene and the synthesis price and complexity. Consequently, not only graphene but also other graphene-based materials are considered very good substitutes of graphene in many applications. In particular, graphene-based materials have been widely used for sensing applications in the last few years due to their high specific surface area, high electronic mobility and low electrical noise. A wide range of chemical sensors, biosensors and gas sensors have been developed using graphene materials.

## 2. Contributions

This Special Issue includes seven works focused on sensors based on diverse technologies for different applications with the common particularity that all of them use graphene materials.

In “Near Room Temperature Light-Activated WS_2_-Decorated rGO as NO_2_ Gas Sensor” [1], Paolucci et al. report how they have exfoliated WS_2_ commercial powders into mono-to-few-layer flakes of WS_2_, and dispersed them with rGO flakes to obtain a WS_2_-decorated rGO. They deposited it on Si_3_N_4_ substrates with platinum finger-type electrodes to build a chemo-resistive NO_2_ sensor that operates at near room temperature conditions and achieves a detection limit of 400 ppb NO_2_ and reproducible gas responses. Cross-sensitivity tests with humid air showed a very low influence of water vapor on the NO_2_ response.

In “NO_2_ and NH_3_ Sensing Characteristics of Inkjet Printing Graphene Gas Sensors” [2], the authors use a different strategy for detecting NO_2_ and NO_3_. Here, the sensitivity of graphene-based chemiresistor gas sensors, fabricated through inkjet printing, is investigated using different concentrations of graphene in the inks. The response of these sensors towards humidity, nitrogen dioxide and ammonia has been characterized, showing that when the sensing layer is not homogeneous and does not cover all the electrode area, the reaction of the sensor towards gases is slower. Another important parameter is the thickness of the graphene film. This work shows that when the layer is too thick, the current flows through independent parallel paths, which brings it to a lower sensitivity. Conversely, if the film is too thin, thermal noise degrades the signal to noise ratio at the output. Moreover, thinner graphene films show a higher sensitivity and faster recovery time.

In “Stress-Insensitive Resonant Graphene Mass Sensing via Frequency Ratio” [3], the applicability of a stretched graphene-based mass sensor via a frequency ratio is presented. To study this device, the authors perform a molecular dynamics simulation. With regard to the square graphene sheet peripherally clamped, the frequencies and the mass-induced frequency shifts of mode11, mode21 and mode22 are analyzed. The simulation results show that absorbed mass in areas with a larger vibration amplitude decreases resonant frequencies more dramatically. Additionally, a strong linear relationship between the frequencies and the square root of stress in graphene was found. The stretched graphene sheet tends to have higher resonant frequencies and higher sensitivities. Compared with the traditional method of mass determination based on the fundamental frequency shift due to the unstable stress in stretched graphene, the proposed method of mass determination via the frequency ratio can achieve a mass resolution of 3.30 × 10^−22^ g. The benefit of stress immunity indicates the great robustness of the proposed sensor against external disturbances in real conditions.

The addition of nanomaterials such as nanoparticles, carbon nanotubes or graphene-based materials may enhance the sensitivity of biosensors. In particular, “Graphene-Based Glycan Biosensor for Electrochemical Label-Free Detection of a Tumor-Associated Antibody” [4] describes the development of a glycan biosensor for the detection of a tumor-associated antibody. This glycan ultrasensitive biosensor is built on an electrochemically activated/oxidized graphene screen-printed electrode. This system is able to selectively detect its analyte with only a minute response after the addition of a control protein, showing high reproducibility.

Although graphene has been widely used as a nano-filler to enhance the conductivity of porous materials, it is still a requirement to prepare graphene-based sponge porous materials by simple and low-cost methods to enhance their mechanical properties and make them have good sensing and capacitive properties. In “Multifunctional Graphene-Based Composite Sponge” [5], a multifunctional graphene-based sponge nanocomposite fabricated using a simple and inexpensive method is presented. The composition of the graphene filler has been deeply studied and optimized. The sponge nanocomposites developed achieve a good sensitivity and electrical response under bending, torsion and compression loads, showing a good performance in flexible sensor and supercapacitor applications.

“Mechanical, Electrical, and Piezoresistive Sensing Characteristics of Epoxy-Based Composites Incorporating Hybridized Networks of Carbon Nanotubes, Graphene, Carbon Nanofibers, or Graphite Nanoplatelets” [6] compares the mechanical, electrical, morphological and piezoresistive characteristics of epoxy-based sensing nanocomposites fabricated with inclusions of hybridized networks of four different carbon nanomaterials: carbon nanotubes, graphene, carbon nanofibers and graphite nanoplatelets.

To finish, “Development of Graphene Quantum Dots-Based Optical Sensor for Toxic Metal Ion Detection” [7] exhaustively reviews the progression of optical sensors for toxic metal ion detection using graphene quantum dots-based materials since they were first introduced. Additionally, the authors also discuss the use of graphene-based materials in surface plasmon resonance-based sensors for metal ion detection.
